# IKKβ inhibitor in combination with bortezomib induces cytotoxicity in breast cancer cells

**DOI:** 10.3892/ijo.2014.2273

**Published:** 2014-01-23

**Authors:** HIROMASA HIDESHIMA, YASUHIRO YOSHIDA, HIROSHI IKEDA, MAYA HIDE, AKINORI IWASAKI, KENNETH C. ANDERSON, TERU HIDESHIMA

**Affiliations:** 1 Department of Medical Oncology, Dana-Farber Cancer Institute, Boston, MA, USA;; 2 Department of Thoracic, Endocrine and Pediatric Surgery, Faculty of Medicine, Fukuoka University, Jonan-ku, Fukuoka;; 3 Department of Internal Medicine, Sapporo Medical University School of Medicine, Chuo-ku, Sapporo 060-8543, Japan

**Keywords:** bortezomib, NF-κB, IKKβ inhibitor, breast cancer

## Abstract

Bortezomib is a proteasome inhibitor with remarkable clinical antitumor activity in multiple myeloma (MM) and is under evaluation in clinical trials in various types of cancer including breast cancer. Although the initial rationale for its use in cancer treatment was the inhibition of NF-κB activity by blocking proteasomal degradation of IκBα, direct evidence indicating inhibition of constitutive NF-κB activity by bortezomib in tumor cells in patients has not yet been reported. Moreover, recent studies have shown that bortezomib activates constitutive NF-κB activity via stimulating the canonical pathway in MM cells. In this study, we first examined protein expression of IκBα after bortezomib treatment. We observed that bortezomib upregulated the phosphorylation and downregulated IκBα protein expression in a dose- and time-dependent manner in MCF7 and T47D cells, associated with phosphorylation of IKKβ. Since IκBα is an inhibitor of nuclear translocation of NF-κB, we further examined alteration of NF-κB activity by bortezomib. Importantly, bortezomib significantly upregulates NF-κB activity in both MCF7 and T47D in a dose-dependent fashion, demonstrated by electrophoretic mobility shift analysis (EMSA). Furthermore, immunocytochemical analysis confirmed enhanced nuclear translocation of p65 NF-κB (RelA) by bortezomib treatment. Supershift assay showed supershifted bands by anti-p65 and -p50 antibodies. Taken together, these results indicate that bortezomib activates the canonical NF-κB pathway in both cell lines. Finally, we demonstrated that IKKβ inhibitor enhanced cytotoxicity, associated with inhibition of NF-κB activity induced by bortezomib in MCF7 and T47D breast cancer cells.

## Introduction

Bortezomib is a reversible 26S proteasome inhibitor which was approved by the Food and Drug Administration in 2003, 2005 and 2008 for the treatment of relapsed/refractory, relapsed and newly diagnosed MM, respectively. The initial rationale to use bortezomib is inhibition of NF-κB activity, since NF-κB plays a crucial role in the pathogenesis in many types of cancer cells, including MM. The NF-κB complex is typically a dimer comprised of different combinations of Rel family proteins, including p65 (RelA), RelB, c-Rel, p50 (NFκB1), and p52 (NFκB2). Previous studies have revealed that NF-κB activity is mediated via two distinct, canonical and non-canonical, pathways (1–4). In the canonical pathway, NF-κB is typically a heterodimer composed of p50 and p65 subunits (5), and its activity is inhibited by association with IκB family proteins (6). Following stimulation by various factors, IκB protein is phosphorylated by IκB kinase (IKK), typically IKKβ. Phosphorylated IκB is subsequently poly-ubiquitinated and degraded by the 26S proteasome (7,8), which allows p50/p65 NF-κB nuclear translocation. Bortezomib inhibits degradation of IκBα and therefore blocks NF-κB activity.

Although bortezomib shows remarkable antitumor activities in preclinical (9–11) and clinical studies (12–14) in MM, in most solid tumor populations, including breast cancer, bortezomib as monotherapy has not shown promising activity (15,16). Bortezomib-based combination therapies have also been conducted using capecitabin (17), pegylated lisosomal doxorubicin (18), docetaxel (19) or paclitaxel (20). In these studies, 15 and 29% response rates were observed in combination with capecitabin and docetaxel, respectively.

However, importantly, recent studies have shown that bortezomib activates canonical NF-κB pathway both *in vitro* and in a human MM cell mouse xenograft model, associated with downregulation of IκBα. Moreover, IKKβ inhibitors augment bortezomib-induced cytotoxicity (21). These results strongly suggest that NF-κB may not be a major target of bortezomib in the treatment of cancer cells. In this study, we therefore examined whether bortezomib also activates NF-κB activity in breast cancer cells, which may, at least in part, account for the insensitivity of these cells to bortezomib. Although constitutive NF-κB activity was low, bortezomib significantly induced the canonical NF-κB pathway, which was blocked by IKKβ inhibitor, associated with enhanced cytotoxicity of bortezomib.

## Materials and methods

### Cells

T47D and MCF7 breast cancer cells as well as RPMI 8226 multiple myeloma cells were obtained from the ATCC (Manassas, VA). T47D and RPMI8226 cells were cultured in RPMI-1640 containing 10% fetal bovine serum (FBS, Sigma Chemical Co., St. Louis, MO), 2 *μ*M L-glutamine, 100 U/ml penicillin and 100 *μ*g/ml streptomycin (Gibco-BRL, Grand Island, NY). MCF7 were cultured in Dulbecco’s modified Eagle’s medium with the above supplements.

### Reagents

Bortezomib was purchased from Toronto Research Chemicals Inc. (North York, ON, Canada). IKKβ inhibitor BMS-345541 was purchased from Calbiochem (San Diego, CA).

### Electrophoretic mobility shift analysis (EMSA)

EMSA was carried out for detection of NF-κB activity, as previously reported (4). Briefly, nuclear extracts from MM cells were obtained using Nuclear Extraction Kit^®^ (Panomics, Fremont, CA). Double-stranded NF-κB oligonucleotide probe (Promega, Madison, WI) were end-labeled with [γ^32^P]ATP (10 mCi/ml, Perkin-Elmer, Boston, MA). Binding reactions containing 0.035 pmol/*μ*l of oligonucleotide and 10 *μ*g of nuclear protein were conducted at room temperature for 30 min in binding buffer (10 mM Tris-HCl, pH 7.5, 50 mM NaCl, 1 mM MgCl_2_, 0.5 mM EDTA, 0.5 mM DTT, 4% glycerol (v/v) and 0.5 *μ*g poly (dI-dC) (Pharmacia, Peapack, NJ). The samples were loaded onto a 4% polyacrylamide gel, transferred to Whatman paper (Whatman International, Maidstone, UK) and visualized by autoradiography. For supershift analysis, 1 *μ*g of anti-p65, RelB, c-Rel (Santa Cruz Biotechnology, Santa Cruz, CA), p50 (Abcam, Cambridge, MA) or p52 (Rockland, Gilbertsville, PA) Abs were incubated for 5 min prior to adding the reaction mixtures.

### Cell proliferation assay

The inhibitory effect of bortezomib, alone or combined with BMS-345541, on cell growth was assessed by measuring 3-(4,5-dimethylthiazol-2-yl)-2,5-diphenyl tetrasodium bromide (MTT, Chemicon International, Temecula, CA) dye absorbance. Cells were pulsed with 10 *μ*l of 5 mg/ml MTT to each well for the last 4 of 24- and/or 48-h cultures, followed by 100 *μ*l isopropanol containing 0.04 N HCl. Absorbance was measured at 570/630 nm using a spectrophotometer (Molecular Devices Corp., Sunnyvale, CA). All experiments were performed 3 times in quadruplicate.

### Immunoblot analysis

MM cells were harvested and lysed using lysis buffer: 50 mM Tris-HCl (pH 7.4), 150 mM NaCl, 1% NP-40, 5 mM EDTA, 5 mM NaF, 2 mM Na_3_VO_4_, 1 mM PMSF, 5 *μ*g/ml leupeptine and 5 *μ*g/ml aprotinin. Whole cell lysates were subjected to SDS-PAGE and transferred to PVDF membrane (Bio-Rad Laboratories, Hercules, CA). The Abs used for immunoblot analysis included: anti-phospho (p)-RIP2 (Ser176), p-IKKα/β (ser176/180), p-p65 (Ser536), p-IκBα (Ser32/36), IκBα and β-catenin (Cell Signaling Technology, Danvers, MA); as well as anti-RIP2, p65, p50, p52, RelB and GAPDH (Santa Cruz Biotechnology) Abs.

### Immunofluorescence

Immunostaining was carried out according to the manufacturer’s protocol. Briefly, T47D cells were cultured for 24 h on Lab-Tek^®^II Chamber Slide System (Thermo Fisher Scientific, Rochester, NY) prior to bortezomib treatment. T47 cells were then treated with 10 nM Bortezomib for 16 h, fixed with 2% formaldehyde-PBS and 100% methanol. After blocking with 5% rabbit serum-PBS for 1 h, slides were incubated overnight with anti-p65 NF-κB Ab (Cell Signaling Technology, Danvers, MA). Cells were then washed and incubated with fluorescence in isothiocyanate-conjugated goat anti-rabbit IgG. Slides were analyzed using Yokogawa spinning disk confocal/TIRE system with Nikon inverted Ti microscope.

### Statistical analysis

Statistical significance of differences observed in drug-treated versus control cultures was determined using the Wilcoxon signed-rank test. The minimal level of significance was p<0.05.

## Results

### MCF7 and T47D cells are relatively resistant to bortezomib treatment

**Figure 1.**
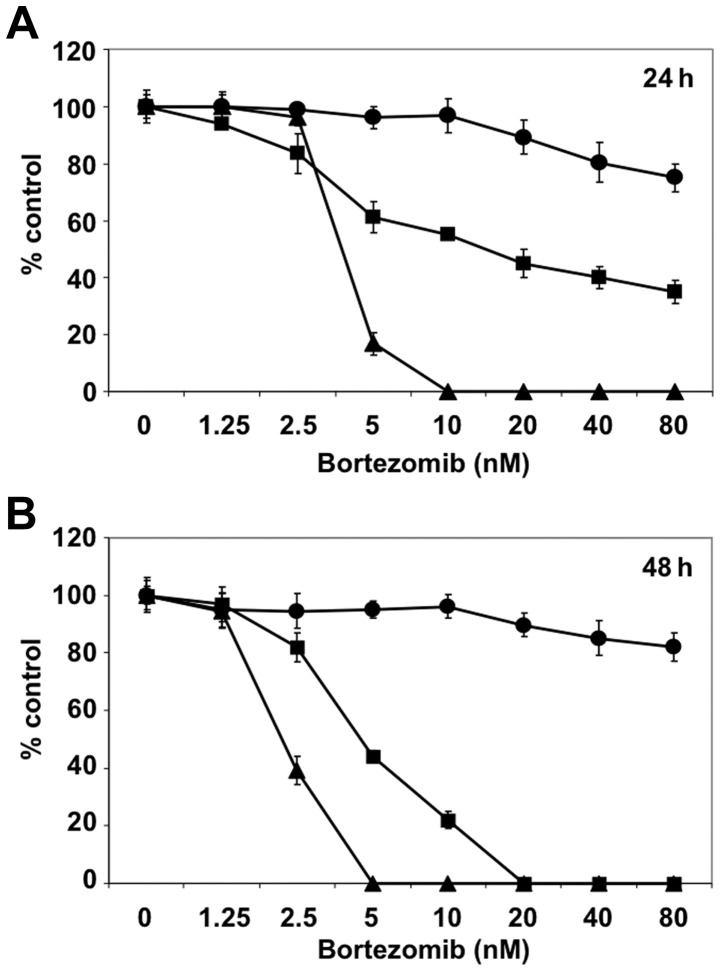
Breast cancer cell lines are relatively resistant to bortezomib treatment. MCF7 (●), T47D (▪) and RPMI 8226 (▴) cells were cultured with bortezomib (1.25–80 nM) for (A) 24 and (B) 48 h, and cell growth was assessed by MTT assay.

We first examined sensitivity of MCF7 and T47D cells to bortezomib treatment. MCF7 and T47D cells were cultured in the presence of different concentration of bortezomib (up to 80 nM) for 24 (Fig. 1A) and 48 h (Fig. 1B). RPMI8226 MM cells were employed as positive control of bortezomib treatment. Compared to RPMI8226, MCF7 and T47D cells were relatively resistant to bortezomib. Especially, bortezomib could not reach the IC_50_ growth inhibition dose in MCF7 cells in this setting.

### Bortezomib downregulates the IκBα protein

**Figure 2.**
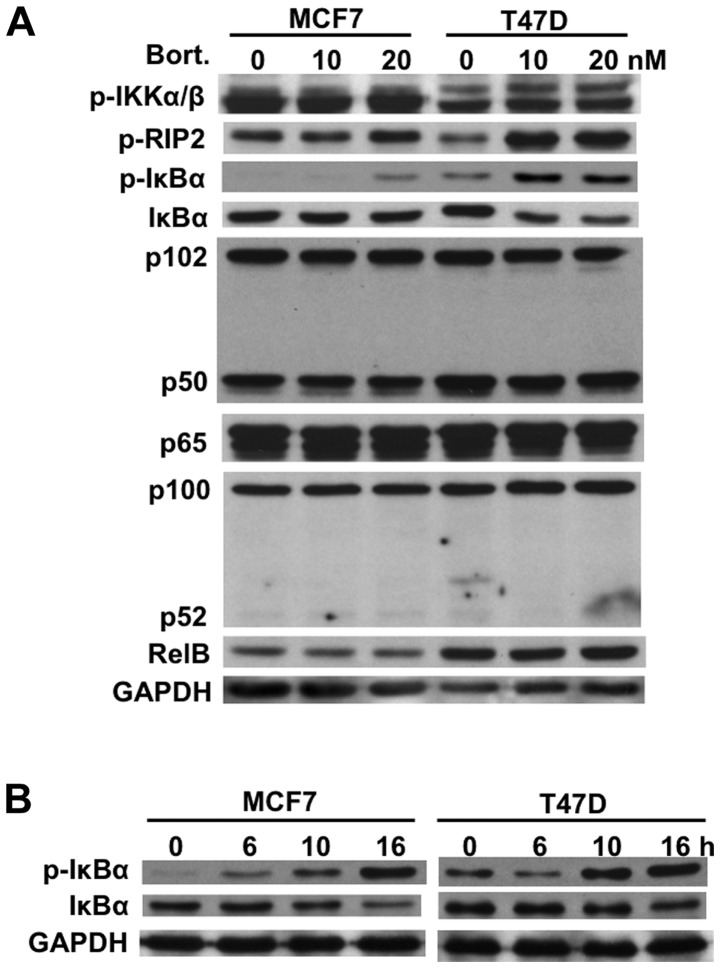
Bortezomib downregulates IκBα expression. (A) MCF7 and T47D cells were cultured with bortezomib (10 and 20 nM) for 8 h. Whole cell lysates were subjected to western blot analysis using indicated antibodies. (B) MCF7 and T47D cells were cultured with bortezomib (10 nM) for the indicated time periods. Whole cell lysates were subjected to western blot analysis using indicated antibodies.

We next examined expression of IκBα and other Rel family member proteins (p50, p52, p65, RelB) in both MCF7 and T47D cells before and after bortezomib treatment. Similar to MM cells (21), bortezomib induced phosphorylation and downregulation of IκBα in both MCF7 and T47D cells after 8-h treatment without alteration of other Rel family member proteins. Interestingly, this effect was more pronounced in T47D cells than in MCF7 cells (Fig. 2A). However, time-dependent study demonstrated that phosphorylation and downregulation of IκBα were similarly observed in both cell lines after 16-h treatment with bortezomib (Fig. 2B). These results strongly suggest bortezomib may activate the canonical NF-κB activity.

### Bortezomib triggers NF-κB activation associated with enhanced p65 (RelA) nuclear translocation

**Figure 3.**
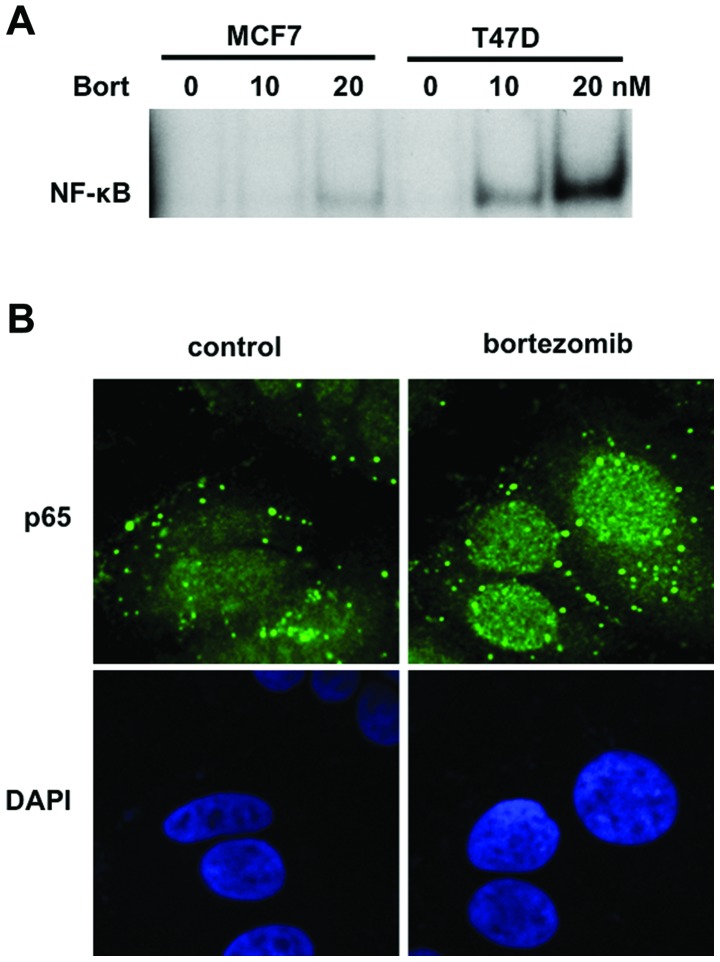
Bortezomib triggers NF-κB activation associated with enhanced p65 (RelA) nuclear translocation. (A) MCF7 and T47D cells were cultured with increasing doses of bortezomib (10–20 nM) of bortezomib or control media for 12 h. Nuclear protein was extracted and subjected to EMSA. (B) T47D cells were cultured with bortezomib (10 nM) or control media for 12 h. Cells were subjected to immunostaining using anti-p65 NF-κB (green) and DAPI (blue) followed by confocal microscopic analysis.

Since IκBα is an inhibitor of nuclear translocation of p50/p65 heterodimer, we next examined whether bortezomib triggered NF-κB activation in breast cancer cells. To obtain direct evidence showing NF-κB activation by bortezomib, we carried out EMSA. Consistent with downregulation of IκBα, bortezomib markedly enhanced NF-κB activity in a dose-dependent fashion in MCF7 and T47D cells (Fig. 3A). We further examined nuclear p65 expression in T47D cells by immunocytochemistry and confirmed that bortezomib markedly enhanced nuclear translocation of p65 (Fig. 3B). These results indicated that NF-κB was activated by bortezomib treatment in breast cancer cells.

### Bortezomib activates the canonical NF-κB pathway in breast cancer cell lines

**Figure 4.**
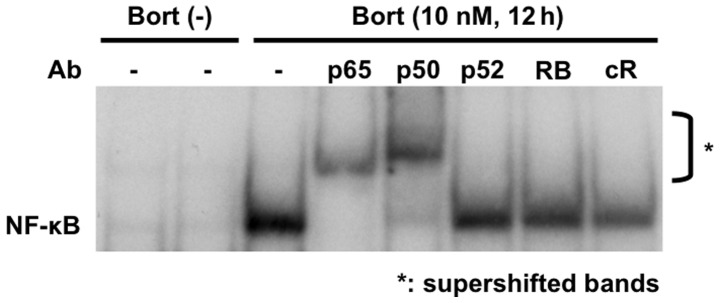
Bortezomib activates the canonical NF-κB pathway. T47D cells were cultured with bortezomib (10 nM) or control media for 12 h. Nuclear extracts from the cells were subjected to supershift assay, using anti-p65, anti-p50, anti-p52 and anti-RelB antibodies.

NF-κB activation is mediated via both canonical and non-canonical pathways, and we further examined whether NF-κB activation by bortezomib was solely via canonical NF-κB activation, since IκBα is a major inhibitor of p50/65 nuclear translocation. Supershift assays confirmed that bortezomib triggered canonical NF-κB activation, evidenced by markedly enhanced supershifted bands in the presence of anti-p65 and p50, but not p52 or RelB (RB) Abs in T47D cells (Fig. 4). This result, strongly indicating that bortezomib triggers activation of the canonical NF-κB pathway.

### Inhibition of IKKβ blocks bortezomib-induced IκBα down-regulation and NF-κB activation

**Figure 5.**
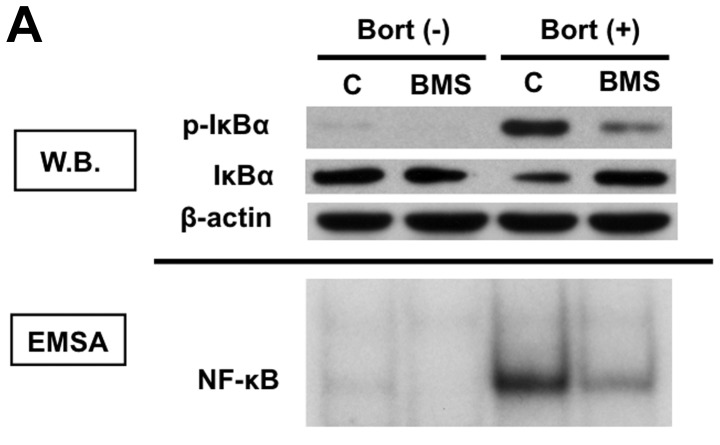

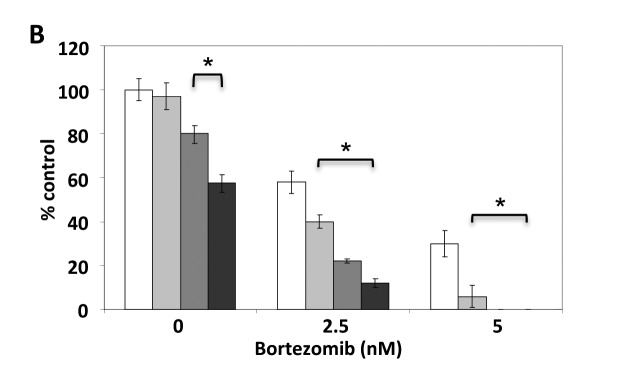
Inhibition of IKKβ blocks bortezomib-induced IκBα downregulation and NF-κB activation. (A) T47D cells were cultured with bortezomib (10 nM) or control media in the presence or absence of BMS-345541 (10 *μ*M) for 12 h and nuclear extracts were subjected to EMSA. Cytoplasmic protein was also immuoblotted with p-IκBα, IκBα and β-actin. (B) T47D cells were cultured with bortezomib (2.5 and 5 nM) in the presence of 2.5 *μ*M (light grey), 5 *μ*M (dark grey) or 10 *μ*M (black) of BMS-345541 for 24 h, and cell growth was assessed by MTT assay. ^*^p<0.01 vs control.

Since bortezomib-triggered NF-κB canonical activation is mediated via phosphorylation and downregulation of IκBα, we next examined whether inhibition of upstream molecule blocked bortezomib-induced NF-κB activation. T47D cells were cultured with bortezomib in the presence and absence of IKKβ inhibitor BMS-345541. BMS-345541 inhibited both phosphorylation and protein expression of IκBα (Fig. 5A, upper panel). Importantly, NF-κB activation induced by bortezomib was completely blocked by BMS-345541 (Fig. 5A, lower panel), suggesting that activation of IKKβ plays a key role in bortezomib-induced NF-κB activation. Moreover, inhibition of canonical NF-κB activity by BMS-345541 enhanced bortezomib-induced cytotoxicity in T47D cells (Fig. 5B).

## Discussion

NF-κB is a transcriptional factor of the Rel family proteins, including p65 (RelA), RelB, c-Rel, p50 (NF-κB1) and p52 (NFκ-B2), which regulates cell proliferation, anti-apoptosis and cytokine secretion in many cancers (22). In canonical pathway, NF-κB is typically a heterodimer composed of p50 and p65 subunits and constitutively present both in the cytosol and nucleus. In the cytosol, p50/p65 nuclear translocation is blocked by IκB family inhibitors; IκBα therefore has a crucial role in regulating NF-κB activation (23). Upon stimulation by various types of growth factors and cytokines (i.e., TNFα), IκBα is phosphorylated by the upstream molecules, IκB kinases (IKKs). IκBα is subsequently polyubiquitinated and degraded by proteasome, allowing nuclear translocation of p50/65, where it binds to specific DNA sequences in the promoters of target genes.

Bortezomib is a 26S proteasome inhibitor initially used in MM treatment based upon expectation that bortezomib could inhibit NF-κB activity by preventing proteasomal degradation of IκBα. It demonstrates remarkable anti-MM activities in both preclinical (9–11) and clinical (12–14) studies, and was approved by FDA in 2003 for therapy of relapsed refractory MM, in 2005 for treatment of relapsed MM, and in 2008 for initial treatment in MM. Although extensive molecular-based studies have been done in MM, inhibition of constitutive NF-κB activity by bortezomib has not been shown in either preclinical or clinical studies. However, in most solid tumors, including breast cancer, bortezomib, as monotherapy, has not shown promising antitumor activity (15,16). Importantly, recent studies have shown that bortezomib activates constitutive NF-κB in endothelial cell carcinoma cells (24) and in primary tumor cells from MM patients (21,25), suggesting that inhibition of NF-κB does not solely account for its antitumor activities. In this study, we therefore examined whether bortezomib also modulates constitutive NF-κB activity regulating cell proliferation and anti-apoptosis in breast cancer cells.

To determine the optimal dose of bortezomib to assess NF-κB activity, we first examined sensitivity of MCF7 and T47D to bortezomib treatment and observed that breast cancer lines, especially MCF7, were relatively resistant to bortezomib. Previous studies have shown that heat shock protein 27 (26,27) and proteasome subunit β5 (PSMB5) gene mutation and overexpression of PSMB5 protein decrease sensitivity to bortezomib in MM and myelomonocytic THP1 cells, respectively (28). However, the mechanisms of action decreasing the sensitivity to bortezomib in these breast cancer cell lines remain unclear.

It has been shown that bortezomib inhibits proteasomal degradation of IκBα induced by cytokines (i.e., TNFα) (29); however, IκBα was downregulated in both cell lines by bortezomib treatment, associated with enhanced phosphorylation in breast cancer cells. These results are similar to previous studies observed in MM cells (21). Since IκBα regulates the canonical NF-κB pathway, we further examined NF-κB activity in MCF7 and T47D cells after bortezomib treatment. Although constitutive NF-κB activity was extremely low, bortezomib markedly enhanced NF-κB activity in a dose-dependent fashion. This result was consistent to down-regulated protein level of IκBα by bortezomib. Interestingly, bortezomib-induced NF-κB activity in T47D was more significant than that in MCF7 cells. As described above, MCF7 cells are more resistant to bortezomib compared to T47D, these results suggested that NF-κB activation may not be totally responsible for resistance to bortezomib in these breast cancer cell lines.

Since NF-κB mediates cell survival and progression of disease in breast cancer (30,31), we hypothesized that the blockade of bortezomib-induced canonical NF-κB activation could enhance its growth inhibitory effect. Indeed, previous study has shown that doxorubicin activates the NF-κB and that inhibition of IKK sensitizes breast cancer cells to bortezomib (32). As expected, IKKβ inhibitor almost completely blocked bortezomib-induced NF-κB activation and significantly augmented its cytotoxicity. Taken together our results provide the preclinical framework for combination strategy of bortezomib with other agents inhibiting IKKβ.
